# Statistical Modelling of Temperature and Moisture Uptake of Biochars Exposed to Selected Relative Humidity of Air

**DOI:** 10.3390/bioengineering5010013

**Published:** 2018-02-09

**Authors:** Luciane Bastistella, Patrick Rousset, Antonio Aviz, Armando Caldeira-Pires, Gilles Humbert, Manoel Nogueira

**Affiliations:** 1 Faculty of Mining and Environmental Engineering—Femma, Federal University of South and Southwest of Pará, Marabá 68507-590, PA, Brazil; batistella.luciane@gmail.com (L.B.); antonioaaviz@gmail.com (A.A.); 2 Joint Graduate School of Energy and Environment, Center of Excellence on Energy Technology and Environment-KMUTT, Bangkok 10140, Thailand; 3 CIRAD, UPR BioWooEB, F-34398 Montpellier, France; 4 Department of Mechanical Engineering, University of Brasilia—UnB, Campus Universitário Darcy Ribeiro, S/N, Asa Norte, Brasília 70910-900, DF, Brazil; armandcp@unb.br; 5 FerroPem, R & D Department, F-73025 Chambéry, France; gilles.humbert@ferroglobe.com; 6 Faculty of Mechanical Engineering—Fem, Federal University of Pará, Belém 66075-900, PA, Brazil; mfmn@ufpa.br

**Keywords:** biochars, moisture uptake, statistical modelling

## Abstract

New experimental techniques, as well as modern variants on known methods, have recently been employed to investigate the fundamental reactions underlying the oxidation of biochar. The purpose of this paper was to experimentally and statistically study how the relative humidity of air, mass, and particle size of four biochars influenced the adsorption of water and the increase in temperature. A random factorial design was employed using the intuitive statistical software Xlstat. A simple linear regression model and an analysis of variance with a pairwise comparison were performed. The experimental study was carried out on the wood of *Quercus pubescens*, *Cyclobalanopsis glauca*, *Trigonostemon huangmosun*, and *Bambusa vulgaris*, and involved five relative humidity conditions (22, 43, 75, 84, and 90%), two mass samples (0.1 and 1 g), and two particle sizes (powder and piece). Two response variables including water adsorption and temperature increase were analyzed and discussed. The temperature did not increase linearly with the adsorption of water. Temperature was modeled by nine explanatory variables, while water adsorption was modeled by eight. Five variables, including factors and their interactions, were found to be common to the two models. Sample mass and relative humidity influenced the two qualitative variables, while particle size and biochar type only influenced the temperature.

## 1. Introduction

Spontaneous combustion has long been recognized as a fire hazard in stored coal and fires usually beginning as “hot spots” deep within the stockpile. Understanding the mechanisms by which carbon-based products get heated to the critical temperature is very important to suppress self-ignition, and ensure secure storage, transport, and handling. On the other hand, self-ignition may also be useful in combustion processes if it occurs under controlled conditions. Therefore, self-ignition can be categorized as a favorable or an unfavorable process which can be controlled by managing the desired parameters. 

New experimental techniques, as well as modern variants on venerable methods, have recently been employed to investigate the fundamental reactions underlying auto-ignition in great detail [1]. A requirement for self-ignition to occur is that the material is sufficiently porous and reactive so that adequate fuel and oxygen are available throughout the whole self-heating process. According to Miura [2], the initial conditions for coal self-heating include many factors which can be divided into two main types: the properties of coal (intrinsic factors) and the environment/storage conditions (extrinsic factors). Heating results from some chemical and/or physical processes occurring within the material and this phenomenon is mainly attributed to exothermic processes such as low temperature oxidation, microbial metabolism, adsorption-desorption of water, and air oxidation with a production of undissipated energy [3,4]. The first advances in self–heating investigations related to air relative humidity were attributed to Davis, who studied the effect of moisture content on the spontaneous combustion of coal using an adiabatic calorimeter. He compared the heat produced by coal in contact with dry and saturated oxygen and showed that the spontaneous combustion started at 70 °C [5]. In the 1960s, Stott confirmed these results and proposed differential equations describing the high-temperature oxidation of coal [6]. Other recent studies have been carried out to clarify the mechanism of low-temperature oxidation of coal and showed that this process is in general very slow compared to air moisture uptake [7,8]. A literature review has been made on present theories and methods for the prediction of spontaneous ignition and has mainly focused on engineering models and small-scale methods [9]. 

The tendency to self-heating is also dependent on material size, so no quantification of a material’s self-ignition hazard is possible without incorporating system size and ambient temperature [10]. Studying charcoal briquettes self-ignition, these authors concluded that a temperature of at least 121 °C is required for self-ignition to occur in the largest commercially available bag size, 9 kg. No information and no correlation have been provided for small size samples (g). The effect of particle size with a diameter in the range 2–50 mm has been studied in a large-scale apparatus and has shown that the spontaneous heating of coal leads to flaming combustion below a certain critical range [11]. The liability of spontaneous combustion of lignite increases with decreasing particle size, increasing moisture content of the coal, and decreasing humidity of the air [12]. The ignition delay of a biomass packed-bed has also been studied and showed an increase with fuel properties such as moisture content and particle size, while it decreased with process conditions such as gas velocity and temperature [13]. The information with a direct temperature increase measurement is expected to be an index to estimate the propensity to spontaneous combustion. The adsorption of water vapor on the sample has been shown to play a crucial role in raising the sample temperature over the critical self-ignition temperature [14].

The purpose of this work was to measure the adsorption rate of water vapor and temperature change of woody and non-woody biochars under various relative humidity conditions naturally occurring in arid, semi-arid, and humid climates. The role of sample size on spontaneous heating was also investigated based on experiments and statistical analyses.

## 2. Material and Method

### 2.1. Material

The four types of biochar used in this study represent major feedstocks and local common bio-reducers in Yunnan Province in China. These are *Quercus pubescens* (Qp), *Cyclobalanopsis glauca* (Cg), *Trigonostemon huangmosun* (Th), and *Bambusa vulgar* (B), which are mainly hardwood except for bamboo which is a non woody biomass. All selected biomass materials were pyrolysed at 500 °C and held for 60 min in order to be roughly similar to the industrial operating conditions of the factory [15]. The proximate analyses followed the standard procedure of the American Society (ASTM D5142). The elemental composition of C, H, and N content was determined using a Thermo FlashEA 1112 Elemental Analyzer (Thermo Fisher Scientific Inc., Waltham, MA, USA) according to the European standard XP CEN/TS 15104 and ASTM D5373 for solid biofuels and charcoals, respectively. The higher heating value (HHV) was experimentally determined with a calorimeter LECO AC350 (LECO Corporation, Saint Joseph, MI, USA). The BET method was applied to provide a precise specific surface area with Belsorp-max Bel Japan equipment (MicrotracBEL Corp., Osaka, Japan). This gear is designed for a wide range adsorption isotherm for surface area and pore size distribution analysis. It can measure adsorption isotherms from relative pressure as low as 1 × 10^−8^ (N2 at 77 K, Ar at 87 K), using a 13.3 Pa pressure transducer. The nitrogen adsorption-desorption of the samples was measured at −196 °C. Prior to the measurements, the samples were degassed at 150 °C for 1 h. The properties of the experimental samples are shown in Table 1. 

### 2.2. Temperature Measurement

**Figure 1:**
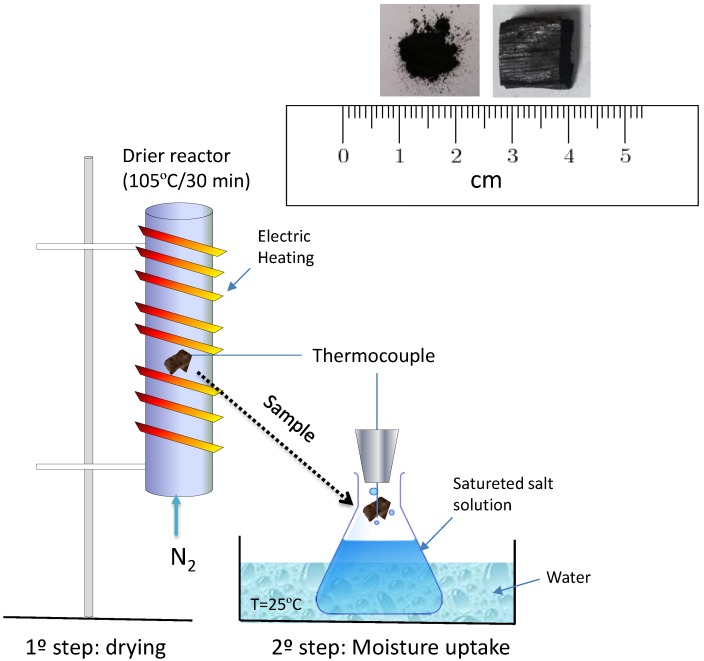
Schematic of experimental setup used for the measurement of temperature change of biomass on exposure to stationary atmosphere (adapted from [2]).

**Figure 2:**
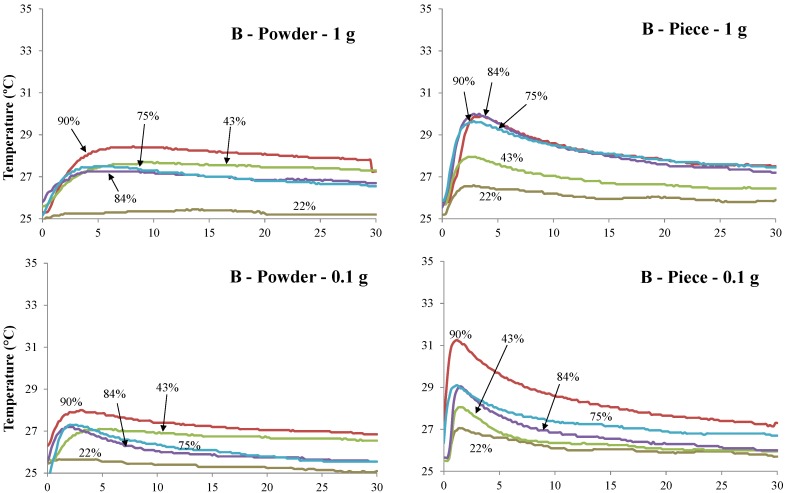

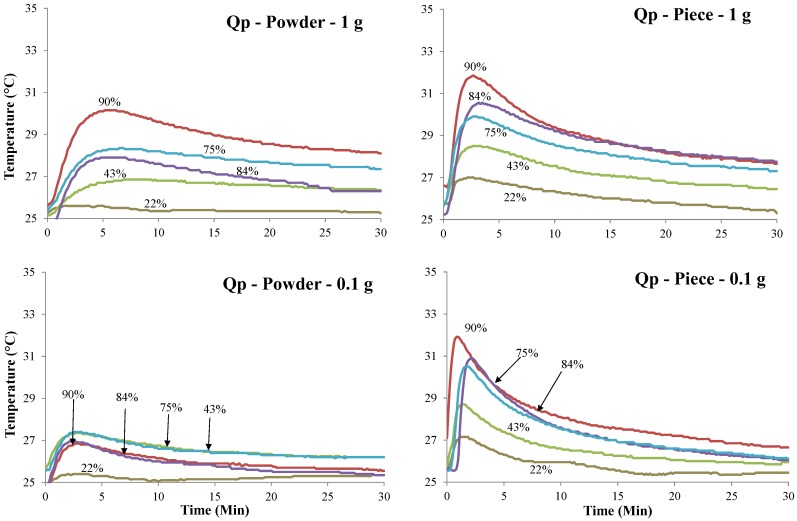
Temperatures (°C) measurement vs. time (min) of *Bamboo* (B) and *Quercus pubescens* (Qp) biochars for powder and piece and 0.1 and 1 g, respectively, exposed to the five selected air humidity (%) conditions.

The adsorption rate of water vapor followed the adapted procedure for coal [2]. Experiments were performed by simulating dry and humid climate conditions where dried samples at room temperature were exposed to the following saturated salt solutions: Potassium acetate (22.6%), potassium carbonate (43.2%), sodium chloride (75.3%), potassium chloride (84.3%), and barium chloride (90.2%) [16]. The solutions were prepared in 1.0 L wide-mouthed glass jars using distilled water and were closed with rubber-join screw caps and stored at 25 °C. In addition, each chamber was equilibrated for one day at 25 °C and immersed in a water bath to guaranty the desired relative humidity. The carbonized biomass samples were processed in the form of pieces and fine particles (250 µm), each with 0.1 g and 1.0 g. The particle samples were placed in a mesh basket (37 µm opening) as a support for all water adsorption experiments. One K-type thermocouple with a 0.5 mm diameter was inserted in the biomass and another close to the basket to measure the changes in temperature during the water adsorption process, as shown in Figure 1. For clarity and accuracy purposes, the temperature records of only *Bamboo vulgaris* and *Quercus pubescens* biochars are displayed in Figure 2. For the other two biochars (Cg and Th), the trend was the same, showing a significant increase in temperature with an increase in relative humidity. Before exposure to saturated salt solutions, samples were dried at 105 °C in a nitrogen stream used as a purging gas at a flow rate of 0.5 L/min. The mass gain due to the adsorption of water vapor was also calculated by drying and weighting the samples after each experiment.

### 2.3. Experimental Protocol

One hundred and sixty (160) assays were conducted, corresponding to eighty (80) treatments and two replicates. The XLSTAT software (Addinsoft company, Paris, France) was used to analyze and reformat data within Excel for statistical analysis. Multiple linear regressions, Analysis of Variance (ANOVA) with stepwise model interactions, and Tukey multiple comparisons were used to relate the two dependent variables that are temperature (*T*) and water adsorption (*W*) with the four independent variables that are biochar species (*B*), relative humidity of air (*RH*), particle size (*S*), and mass of sample (*M*). The two variables, temperature and water adsorption, in response to the experiments, were analyzed and discussed following a (5 × 4^2^) random factorial design. The parameters of the experimental design can be found in Table 2. 

The experimental error had a degree of freedom of 33 and 43 for temperature and water sorption, respectively, as dependent variables. The general model for variance analysis (ANOVA) can be described by the following equation, where each independent variable and their interactions are presented:

Y_ijklr_ = μ + [B_i_ + RH_j_ + S_k_ + M_l_ + (B × RH)_ij_ + (B × S)_ik_ +(B × M)_il_ + (RH × S)_jk_ + (RH × M)_jl_ + (S × M)_kl_ + (B × RH × S)_ijk_ + (B × RH × M)_ijl_ + (B × S × M)_ikl_ + (RH × S × M)_jkl_ + (B × RH × S × M)_ijkl_] + ε_ijkl_

## 3. Results and Discussion

### 3.1. Overall Results

Table 3 and Table 4 provide the average values for water adsorption (*W*) and the temperature (*T*) of biochar pieces and powdered biochar reached when exposed to five different conditions of relative humidity. All samples show a significant increase in temperature. The higher the air humidity value is, the higher the increase in temperature is. This tendency is observed for almost all samples. The highest and fastest increase in temperature is observed for the piece of biochar characterized by the lowest weight (0.1 g) (Figure 2). The temperature profiles of the powdered biochar samples are significantly different from the biochar piece samples and this is independent of the weight of the samples. While the biochar piece samples required around 2 min to reach the peak temperature, the powdered biochar samples required 5 to 8 min under the highest relative humidity conditions. Cg biochar and piece samples reached the highest temperature (6 °C) for a relative humidity of 90.2%.

**Figure 3:**
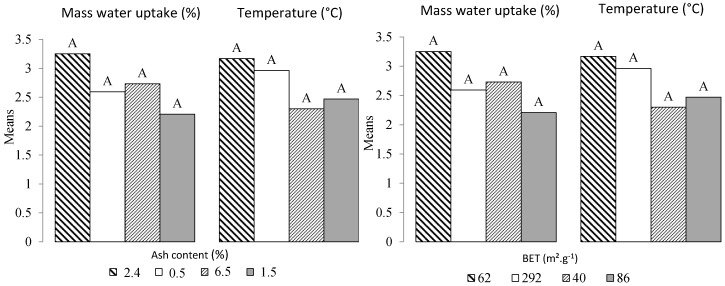
Classification by Tukey’s test for water sorption (*W*) and temperature (*T*) averages versus BET and ash content. The means with the same letter were not significantly different at 5% (α = 0.05).

Concerning the water vapor adsorption for all conditions, all the samples with a mass of 1.0 g show a lower water vapor adsorption capacity than samples with a mass of 0.1 g. The values obtained are more dispersed. However, the global trend shows that more mass of water vapor was adsorbed when the samples were exposed to higher levels of relative humidity. This difference is mainly due to the difference in mass transfer in the samples [2]. The highest amount of water adsorbed is observed for Cg (piece 0.1 g/84.3%), Cg (powder 0.1 g/90.2%), and B (powder 0.1 g/84.3%) with 7%, 6.5%, and 6.2%, respectively. For the powdered biochar samples, the better adsorption capacity can be explained by the larger surface area exposed to outside conditions compared to the piece. The surface area is also an important physical property for self-ignition. A direct correlation between oxygen chemisorption and active surface area has been reported by Zhao [17]. However, this phenomenon does not seem to be correlated to the BET results (Table 1), where (Qp) and (B) biochars with 292 and 40 m^2^·g^−1^, respectively, did not show the strongest and lowest potential for spontaneous combustion, respectively. To confirm this, we performed a one-way balanced analysis of variance. As shown in Figure 3, the Tukey’s HSD (Honestly Significantly Different) test was applied to all pairwise differences between means. As all the combinations shared the same letter, it can be concluded that the BET does not significantly affect water absorption (*W*) and temperature (*T*).

The above discussion shows that the adsorption of water vapor under different relative humidity conditions (Table 3) has the potential to raise the temperature of the samples. However, other biochar-related physical and chemical properties can also affect water sorption and consequently the increase in temperature (Table 4). For example, the mineral content acts as a heat sink [17]. With the increase in mineral content (indicated by increasing ash content), it has been shown that the crossing point of coal temperature increases (CPT), which is used to evaluate the spontaneous combustion of coal. The Crossing-point Temperature (CPT) is the temperature (temperature and corresponding time) at which the increasing coal temperature is equal to the increasing oven temperature within a Temperature-Programmed System (TPS) [18]. These results suggest that Bamboo with 6.5% ash content should be the most subject to spontaneous combustion, while Qp should be the least affected (0.5%). A recent study by [15] showed the opposite. Figure 3 shows no difference related to ash content for the four biochars. In the literature, a lot of models have been developed to predict spontaneous ignition. They were mainly engineering models and small-scale methods requiring producing input data for such models [9]. In the next sections, a statistical analysis based on linear regressions and analysis of variance (ANOVA) is reported, investigating the weight of each independent or explanatory variable according to the model equation presented in the experimental section.

### 3.2. Linear Regression Model

**Figure 4:**
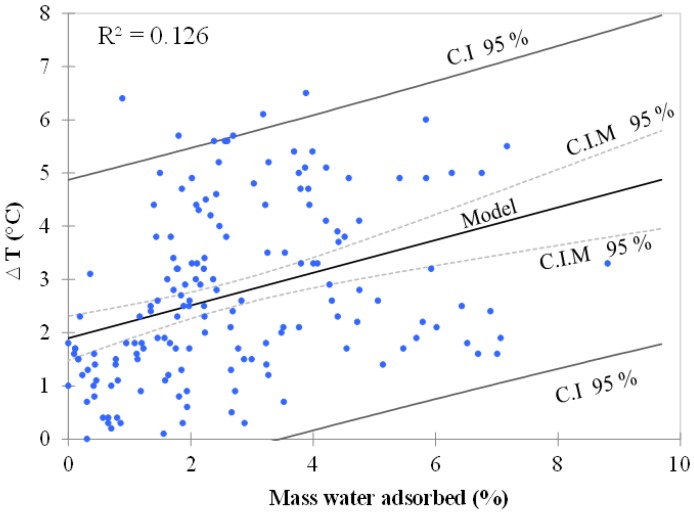
Regression line of temperature (°C) vs. water adsorption (%) with two confidence intervals (C.I) at 95%. The confidence interval on mean (C.I.M) of the prediction for a given value of T is the one closer to the line.

Applying a simple linear regression model based on Ordinary Least Squares (OLS), the objective was to determine how temperature (*T*) varies with water adsorption (*W*) and to verify if a linear model makes sense. The chart from Figure 4 allows us to visualize the data, the regression line (the fitted model), and two confidence intervals at 95%. It can be clearly seen that there is a linear trend, but also a high variability around the line. This high dispersion of results is corroborated by a low R^2^ value (0.126), indicating that only 13% of the variability of the temperature can be explained by water adsorption. The model equation in this case is given by:

∆*T* (°C) = 1.9 + 0.3 × Mass water adsorbed (%).

Several linear regressions were performed to verify if any linear models limited to selected data from each independent variable (*RH*, *B*, *M*, and *S*) could better explain the results obtained. Statistics are summarized in Table 5 and enabled us to determine whether or not the explanatory independent variables bring significant information to the model. Despite generally low R^2^ values for all explanatory variables, the information brought by size (piece), mass (0.1 and 1 g), and type of biochar (Th) is observed to be more significant than the other variables. These variables explain 60, 40, 30, and 17% of the relation between temperature and water adsorption, respectively. Their probabilities corresponding to the F value were found to be lower than 0.0001. If we can partially conclude with confidence that these four independent variables brought a significant amount of information, the linear regression model still shows limitations; using a simple linear regression is not acceptable for the prediction of temperature increase as a function of water adsorption. 

### 3.3. Analysis of Variance (ANOVA)

The ANOVA function was used to find out if the results would differ according to the formula described in Section 2.3 and, if so, which formula is the most effective. A pairwise comparison was performed to be able to run a Tukey’s test, which is generally used in conjunction with an ANOVA to determine which means significantly differ from each other. The test compares the mean of each treatment to the mean of every other treatment. A stepwise method was selected and the statistics corresponding to the different steps were displayed. Finally, the best models for each number or variables with the corresponding statistics and for the criterion chosen were calculated. Table 6 displays the goodness of fit coefficients for the 160 observations, including the R^2^ (coefficient of determination). The two dependent variables display a very low coefficient of variation (<1), indicating a good control over the operating conditions. For both water adsorption and temperature, around 89% of the variability is explained. The remaining 11 percent are hidden in other variables including biochar physical and chemical characteristics, which the model classifies as “random effects”. Given that the probability (Pr) corresponding to the Fisher’s F is lower than 0.0001 for both *W* and *T*, we can conclude that the explanatory variables and their interactions have a significant effect.

To elaborate the two models for each dependent variable (*W* and *T*), the selection process started adding the variable with the largest contribution to the model. If a second variable is such that the probability associated with its “*t*” is less than the “Probability for entry”, it is added to the model. The procedure continues until no more variables can be added. This analysis allowed us to retain eight and nine explanatory variables (Table 7) to predict *W* and *T*, respectively. The cumulative coefficient of determination R² gives a fair idea of how much of the variability of *W* and *T* can be explained by these four qualitative variables and their interactions.

It is observed that the three interactions Size × Biochars, Size × Biochars × RH and Size × Mass × RH do not affect water sorption and temperature, while the variable RH influences them. The same observation is observed with the mass variable (*M*). This means that when explanatory variables are taken independently, they influence experimental results, but if associated, their effects are limited. The two independent variables Size and Biochars, and the second order interaction Biochars × RH, are found to only influence the temperature variable (*T*), while Biochars × Mass, Biochars × Mass × RH and Size × Biochars × Mass × RH, (second, third, and fourth order interactions, respectively) only influence the water sorption variable (*W*). Although the biomasses selected had different morphologic properties, it is noted that these do not affect water adsorption. This observation confirms that the BET area is not correlated to this quantitative variable (*W*). Finally, all the other explanatory variables and their interactions are observed to significantly influence *W* and *T*. For *T*, around 50% of the variations can be explained by the relative humidity variable, while *W* variations can be explained by the interaction “Mass × relative humidity” (R^2^ = 0.48).

**Figure 5:**
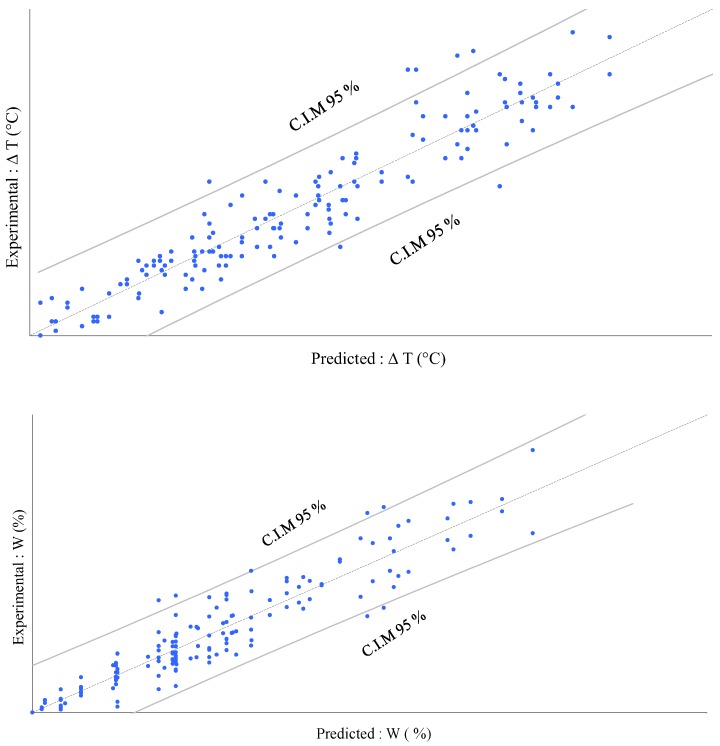
Model predictions vs. experimental results for *T* and *W* with two confidence intervals on mean (C.I.M) of the prediction at 95%.

Figure 5 allows a comparison of the predictions to the experimental values. The confidence limits permit us to identify outliers, as with the regression plot displayed above. The two models bring significant information to explain the experimental results for *W* and *T*. The quite low deviation observed for all points, which remained close to the first bisector line, allow us to conclude that these two models did fit with the experimental results quite well.

The previous conclusions drawn from the means are statistically supported by the pairwise multiple comparisons. Significant information arising from Table 7 was summarized. All the combinations between the levels of the four factors and their interactions were associated to letters after applying the Tukey’s test. This section focuses on the interpretation of all pairwise differences between means. Two level combinations sharing the same letter translate into not being significantly different. Two combinations with no letter in common translate into being significantly different. Attention is paid to factors and interactions that are the most significant according to the models. The following two factors and three interactions were identified: Mass, RH, Size × RH, Mass × RH, and Size × Biochars × Mass.

**Figure 6:**
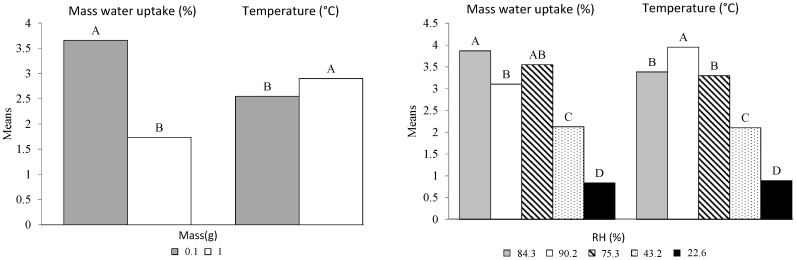
Mass and Relative humidity multiple comparisons according to the Tukey test. The means with the same letter were not significantly different at 5% (α = 0.05).

As a reminder, the variable “Mass” is characterized by two values: 0.1 and 1 g. Figure 6 shows two distinct groups (A and B) for both W and T. Although the difference between the water sorption averages is evident (1.9%), this analysis of variance shows a significant difference for T, despite relatively similar average values (2.55 °C and 2.90 °C). Concerning the explanatory variable RH, the four pairs of categories are found to be different. The two RH pairs 84.3 × 75.3 and 90.2 × 75.3 do not show any significant differences, while the means between 84.2 and 90.2 are significantly different (3.1 and 3.9%). Except for 75.2%, all the other air relative humidity conditions show significant differences with regard to the observed adsorption values. The highest and the lowest values are 3.87 and 0.84% for RH values of 84.4 and 22.6, respectively. The maximum temperature is obtained with the highest relative humidity (3.95 °C) and the minimum temperature with the lowest RH (0.84 °C), as corroborated by the literature [14]. We can conclude that these two factors (Mass and RH) played a significant role in both the two models. 

**Figure 7:**
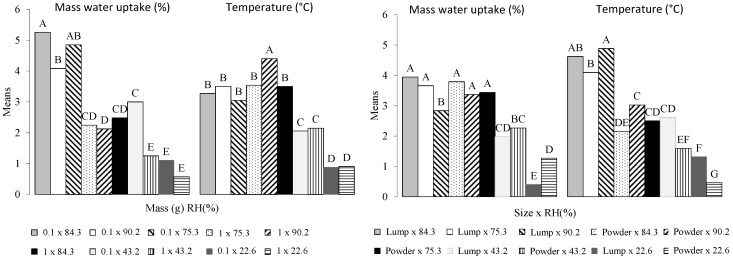
Second order interactions multiple comparisons according to the Tukey test. The means with the same letter were not significantly different at 5% (α = 0.05). Lump = piece.

Figure 7 shows the pairwise multiple comparisons for the second order interactions Size × RH and Mass × RH. For each interaction, all the combinations of levels between the two factors are compared to one another. The number of combinations possible between interactions and variables is 10. The variable Size is characterized by grinded biochar (powder) or biochar piece. The interaction Size × RH shows five and seven groups for water adsorption and temperature, respectively. Water sorption shows five combinations with means that are not significantly different. It concerns mainly high relative humidity values regardless of the sample size. Indeed, the difference appears mainly with the lowest humidity values (22 and 43%). The temperature shows two combinations (Piece × 90.2 and powder × 22.6) with no letter in common. Biochar pieces are more sensitive to relative humidity than powdered biochar, showing a higher average temperature (4.8 °C). This can mainly be attributed to the difference in the mass transfer rates and heat generation related to the sample size [2]. Indeed, it has been demonstrated that the effect of both the temperature overshoot and the pressure is dependent on sample thickness [19].

The combinations associated with the interaction Mass × RH show five and four groups for *W* and *T*. The variable *T* shows lesser variability compared to the Size × RH interaction. Five combinations concerning mainly high relative humidities have the same letter (B). Water sorption is mostly influenced by small mass combined with RH, except when RH is equal to 22.6%. When combined with humidity, the explanatory variable Size mostly influences the temperature, while the variable *M* mostly influences the adsorption of water.

The last common parameter to W and T models is the third order interaction Size × Biochars × Mass. Table 8 gives the average value classified by the Tukey test. The variable *T* presents seven groups and W only four groups. The temperature variable is more sensible to this interaction than the water adsorption variable and can be explained by the factors “biochars” and “Size” only present in the T model (Table 7). The overall results confirm that water adsorption is not correlated to the increase in temperature. Therefore, others elements, not yet identified, are likely involved in the increase in temperature.

## 4. Conclusions

It is well known that the adsorption of water vapour from ambient atmosphere plays a crucial role in raising the temperature of a biochar sample over the critical self-ignition temperature. This study was carried out on the wood of *Quercus pubescens*, *Cyclobalanopsis glauca*, *Trigonostemon huangmosun*, and *Bambusa vulgaris*, and involved five air relative humidity conditions (22, 43, 75, 84, and 90%), two mass samples (0.1 and 1.0 g), and two particle sizes (powder and piece). All experimental results showed a significant increase in temperature with the relative humidity. The highest and fastest increases in temperatures were observed for biochar pieces coupled to the lowest weight (0.1 g). Biochar pieces needed around 2 min to reach the temperature peak; powdered samples needed 5 to 8 min. The global trend showed that a larger mass of water vapor was adsorbed when exposed to a higher relative humidity. All samples with a mass of 1.0 g showed a lower water vapor adsorption compared to samples at 0.1 g. A linear regression model based on the temperature and the water adsorption (W) showed a high dispersion of the results corroborated by a low R^2^ value (0.13). Two models were elaborated for each dependent variable (*W* and *T*) to simulate water adsorption and temperature. Eight and nine qualitative variables and their interactions were selected for *W* and *T*, respectively. Sample mass and relative humidity influenced both *W* and *T*, while particle size and type of biochar mainly influenced the temperature. Thus, these findings are very important not only for all scientific aspects, but also in practical applications. They will allow the creation of tabulations giving recommendations for charcoal cooling and storage considering the season (RH) and the critical size of the samples, and consequently to anticipate cool flame phenomena. 

## Figures and Tables

**Table 1:** Physicochemical characteristics of biochars.

Properties	Biochar			
	Qp	Cg	Tr	B
Proximate analysis (wt %, dry basis)				
Ash	0.5	2.4	1.5	6.5
Volatile matter	12.7	17.4	15.1	15.6
Volatile matter/Ash	25.4	7.3	10.1	2.4
Fixed carbon	86.8	80.2	83.4	77.8
Ultimate analysis (wt %, dry basis and ash free)				
C	89.6	86.3	89.3	82.2
H	2.3	2.3	2.3	1.5
N	0.5	0.3	0.3	0.5
O (by difference)	7.6	11.1	8.1	15.8
H/C	0.02	0.03	0.02	0.02
High heating value (Mj·Kg^−1^)	33.9	32.7	33.8	30.4
BET Surface area (m^2^·g^−1^)	292	62	86	40

**Table 2:** Values of the parameters selected for the experimental design.

Parameters Level	Relative Humidity (%)	Biochars	Particle Size	Mass (g)
1	22.6	*Quercus pubescens* (Qp)	Piece	1
2	43.2	*Cyclobalanopsis glauca* (Cg)	Powder	0.1
3	75.3	*Trigonostemon huangmosun* (Th)	-	-
4	84.3	*Bambusa vulgar* (B)	-	-
5	90.2	-	-	-

**Table 3:** Averaged values for water adsorption (%) from pieces and powdered biochars exposed to different relative humidities of air based on a random factorial design, considering two replicates per test. (d.b. = dry basis).

Sample	Size	Mass (g)	Relative Humidity (%)				
			22.6	43.2	75.3	84.3	90.2
			Moisture Uptake (%) d.b.				
Qp	Piece	0.1	0.14	2.97	5.30	3.95	1.96
		1	0.43	0.28	2.22	3.23	2.20
Cg	Piece	0.1	0.00	3.25	5.43	6.96	4.87
		1	0.13	1.32	3.55	3.49	1.72
Th	Piece	0.1	0.37	2.53	4.46	4.82	2.72
		1	0.35	1.66	1.92	2.28	2.19
B	Piece	0.1	1.20	2.14	4.29	3.77	4.11
		1	0.61	1.72	2.12	3.06	1.42
Qp	Powder	0.1	2.35	4.97	4.02	4.25	5.21
		1	0.65	1.35	1.78	1.26	1.76
Cg	Powder	0.1	2.73	2.83	5.58	6.15	6.50
		1	0.88	1.07	3.23	1.77	3.60
Th	Powder	0.1	0.31	2.36	2.33	5.10	3.77
		1	0.70	1.10	1.00	2.12	2.05
B	Powder	0.1	1.72	2.96	2.37	6.24	3.52
		1	0.83	1.51	1.74	1.81	2.07

**Table 4:** Averaged values for temperature (°C) increasing from pieces and powdered biochars exposed to different relative humidities of air based on a random factorial design, considering two replicates per test.

Sample	Size	Mass (g)	Relative Humidity (%)				
			22.6	43.2	75.3	84.3	90.2
			∆*T* (°C)				
Qp	Piece	0.1	1.60	2.95	5.05	5.25	4.80
		1.0	1.20	2.70	4.25	5.30	5.25
Cg	Piece	0.1	1.40	3.05	4.95	5.25	5.70
		1.0	1.55	2.45	4.75	4.61	6.00
Th	Piece	0.1	1.15	2.45	3.85	4.50	5.30
		1.0	1.15	2.50	3.35	4.20	4.40
B	Piece	0.1	1.10	2.55	2.75	3.40	3.50
		1.0	1.40	2.25	3.8	4.45	4.10
Qp	Powder	0.1	0.55	1.70	1.85	2.10	2.25
		1.0	0.35	1.70	2.75	3.10	4.55
Cg	Powder	0.1	0.65	1.40	2.35	2.00	2.55
		1.0	0.65	1.80	5.35	2.00	4.95
Th	Powder	0.1	0.35	1.40	0.90	2.00	2.25
		1.0	0.60	1.70	1.60	2.90	2.85
B	Powder	0.1	0.20	0.95	2.70	1.65	1.65
		1.0	0.35	2.10	2.50	1.45	3.15

**Table 5:** Summary statistics for the linear regression model of *T* (°C) vs. *W* (%) for each qualitative variable. P. = piece; Pow. = powder.

	Biochars				Relative Humidity (%)					Mass (g)		Size	
	Qp	Cg	Th	B	22.6	43.2	75.3	84.3	90.2	0.1	1.0	P.	Pow.
Min	0.31	0.40	0.00	0.10	0.00	0.50	0.92	1.27	1.23	0.02	0.20	0.83	0.04
Max	6.00	6.53	6.10	4.70	1.87	3.50	6.02	5.51	6.53	6.54	6.45	6.52	5.73
Average	3.03	3.20	2.51	2.30	0.90	2.10	3.31	3.43	4.07	2.62	2.92	3.55	1.93
Std.dev.	1.70	1.80	1.50	1.24	0.50	0.70	1.43	1.48	1.52	1.61	1.65	1.51	1.24
R^2^	0.05	0.15	0.29	0.06	0.20	0.01	0.05	0.01	0.11	0.17	0.59	0.42	0.07
Pr > F	0.1590	0.015	<0.0001	0.1240	0.0100	0.6430	0.2010	0.8910	0.070	<0.0001	<0.0001	<0.0001	0.0150

**Table 6:** Summary statistics for the experimental factorial design performed considering a mean of two replicates.

Variable	Minimum	Maximum	Mean	Std. Dev.	R^2^	F	Pr > F
Mass water uptake (%)	0.00	8.81	2.70	1.83	0.88	19.31	<0.0001
∆*T* (°C)	0.00	6.50	2.72	1.60	0.89	30.72	<0.0001

**Table 7:** Statistics synthesis for explanatory variables and their interactions. R^2^ values are cumulated. *p*-values < 0.0001 = significant; ns = not significant.

		Model: *W* (%)	Model: ∆*T* (°C)
Size	R^2^		0.72
	F		274.83
	Pr > F	ns	<0.0001
Biochars	R^2^		0.77
	F		18.85
	Pr > F	ns	<0.0001
Mass (g)	R^2^	0.61	0.85
	F	259.86	13.95
	Pr > F	<0.0001	0.0000
RH (%)	R^2^	0.68	0.48
	F	85.11	136.22
	Pr > F	<0.0001	<0.0001
Size × Biochars		ns	ns
Size × Mass (g)	R^2^		0.87
	F		17.76
	Pr > F	ns	<0.0001
Size × RH (%)	R^2^	0.88	0.80
	F	3.19	9.63
	Pr > F	0.0160	<0.0001
Biochars × Mass (g)	R^2^	0.73	
	F	14.26	
	Pr > F	<0.0001	ns
Biochars × RH (%)	R^2^		0.84
	F		3.67
	Pr > F	ns	<0.0001
Mass (g) × RH (%)	R^2^	0.47	0.89
	F	11.13	2.89
	Pr > F	<0.0001	0.0250
Size × Biochars × Mass (g)	R^2^	0.77	0.88
	F	9.74	3.31
	Pr > F	<0.0001	0.0220
Size × Biochars × RH (%)		ns	ns
Size × Mass (g) × RH (%)		ns	ns
Biochars × Mass (g) × RH (%)	R^2^	0.81	
	F	3.37	
	Pr > F	0.0000	ns
Size × Biochars × Mass (g) × RH (%)	R^2^	0.87	
	F	4.12	
	Pr > F	<0.0001	ns

**Table 8:** Classification by Tukey’s test in decreasing order for *T*. For each group, the means with the same letter were not significantly different at 5% (α = 0.05).

Parameters	Mass Water Sorbed (%)	∆ *T* (°C)
Piece × Cg × 0.1	4.10 ^ab^	4.07 ^a^
Piece × QP × 0.1	2.86 ^c^	3.93 ^a^
Piece × Cg × 1	1.86 ^d^	3.89 ^a^
Piece × QP × 1	1.86 ^d^	3.66 ^ab^
Piece × Th × 0.1	3.00 ^c^	3.45 ^abc^
Piece × B × 1	1.86 ^d^	3.20 ^bcd^
Piece × Th × 1	1.86 ^d^	3.16 ^bcd^
Powder × Cg × 1	1.60 ^d^	2.73 ^cd^
Piece × B × 0.1	3.10 ^bc^	2.66 ^cde^
Powder × QP × 1	1.60 ^d^	2.50 ^de^
Powder × B × 1	1.60 ^d^	2.04 ^ef^
Powder × Th × 1	1.60 ^d^	2.00 ^ef^
Powder × Cg × 0.1	4.75 ^a^	1.98 ^ef^
Powder × QP × 0.1	4.31 ^a^	1.75 ^fg^
Powder × B × 0.1	4.37 ^a^	1.29 ^g^
Powder × Th × 0.1	2.74 ^c^	1.25 ^g^
